# Supplementary material for: Content-rich biological network constructed by mining PubMed abstracts
Source: BMC Bioinformatics. 2004 Oct 8;5:147. doi: 10.1186/1471-2105-5-147 (PMC528731; doi:10.1186/1471-2105-5-147)
Supplement: Additional File 2 — The original results of the above study (non-essential files are deleted to keep the file size under the limit set by BMC bioinformatics). [file 1471-2105-5-147-S2.bz2 › chilibotAdditionalFile2/dip10/618ID8736558E11002/html/TXN_APEX1.html]

 


 **TXN** and **APEX1** 
  
Found 454 abstracts in PubMed, retrieved 10.  
 

 What does Google say? 
 PDF only 
| .edu only 

---

**Interactive relationship** (e.g. stimulation, inhibition, etc)

**Inhibitory relationship**- Trx  [ **TXN** ]  1 transfected MCF 7 cells showed decreased Nrf 2 PMF 1 DNA  [ **APEX1** ]  binding without a change in Nrf 2 or PMF 1 protein expression.  Ref: 12804587 Biochem Biophys Res Commun, 2003
- Recombinant Bm TRX  [ **TXN** ]  1 had thiodisulfide reductase activity, as measured by the reduction of insulin, and protected DNA  [ **APEX1** ]  from the nicking activity of oxygen radicals.  Ref: 12819103 Infect Immun, 2003
**Stimulatory relationship**- Therefore, increases in nuclear Trx  [ **TXN** ]  and Ref 1  [ **APEX1** ]  during aging may result in the up regulation of redox sensitive transcription factors, such as NF kappaB or AP 1, via the interaction of Ref 1  [ **APEX1** ]  and Trx  [ **TXN** ]  in a redox dependent manner.  Ref: 12742531 Exp Gerontol, 2003
**Neutral relationship**- Myocardial TRX  [ **TXN** ]  was upregulated in myocarditis and cardiomyopathies with active necrotic stage associated with DNA  [ **APEX1** ]  damage, which may reflect the oxidative stress overload in hemodynamically uncontrolled status.  Ref: 12870673 Mol Cell Biochem, 2003

**Non-interactive relationship** (e.g. studied together, co-existance, homology, etc.)

- FACS analysis of DNA  [ **APEX1** ]  fragmentation showed that the TRX  [ **TXN** ]  gene suppressed hepatocyte apoptosis.  Ref: 12878175 Biochem Biophys Res Commun, 2003
- In contrast, we also found that the nuclear translocation of the redox regulators, Trx  [ **TXN** ]  and Ref 1  [ **APEX1** ] , increase with age, which was suppressed in CR rats.  Ref: 12742531 Exp Gerontol, 2003
